# US and CT of the Liver after Electric Shock

**DOI:** 10.1155/2016/9846357

**Published:** 2016-02-23

**Authors:** Amela Sofić, Nermina Bešlić, Alma Efendić, Aladin Čarovac, Jusuf Šabanović, Elma Jahić, Melika Bukvić, Fikreta Krakonja, Jana Kupusović

**Affiliations:** ^1^Institute of Radiology, University Clinical Center Sarajevo, Sarajevo, Bosnia and Herzegovina; ^2^Institute of Nuclear Medicine, University Clinical Center Sarajevo, Sarajevo, Bosnia and Herzegovina; ^3^Department of Radiology, Cantonal Hospital Zenica, Zenica, Bosnia and Herzegovina; ^4^Clinic of Abdominal surgery, University Clinical Center Sarajevo, Sarajevo, Bosnia and Herzegovina; ^5^Institute of Gastroenterology and Hepatology, University Clinical Center Sarajevo, Sarajevo, Bosnia and Herzegovina

## Abstract

Liver injuries caused by high voltage electricity are rare and result in high mortality and morbidity. They are produced by the resistance to the passage of electrical current through the tissue, which creates heat that leads to coagulation necrosis and rupture of the cell membrane. We present a case of an electrical injury to the liver, diagnosed by ultrasound and CT in a 39-year-old man who presented with skin burns on his right hand and right hemiabdomen. Injuries occurred after the contact with 220 kV high voltage electricity.

## 1. Introduction

Electrical burns are associated with high morbidity and mortality. They are rare and account for approximately 5% of patients admitted to the major burn centres.

Electrical lesions occur due to high voltage currents (>1000 V, 50 Hz) mostly at the workplace when a worker comes into direct contact with the source of energy or indirectly via a conductive material and equipment [1].

Electrical burn is a result of heat and electricity that passes through the tissue, causing coagulation necrosis and rupture of the cell membrane. The resistance of the tissue to the passage of electrical current varies depending on the tissue and is lower for nerves and higher for fat and bones. An electrical current of the same intensity can cause variable damage depending on the sensitivity of each individual and the quality of the medical treatment after injury [2]. First artificial electricity related injuries were reported 300 years ago. The first recorded accidental electrocution occurred in 1879 when a stage carpenter in Lyon, France, touched a 250-volt AC generator [3]. Visceral injuries are rare in victims of electrical burns. Reported cases show that the organs most frequently affected are the colon and small intestine, while less frequently involved organs are heart, esophagus, stomach, pancreas, liver, gallbladder, lung, and kidney [4, 5].

## 2. Case Report

**Figure 1:**
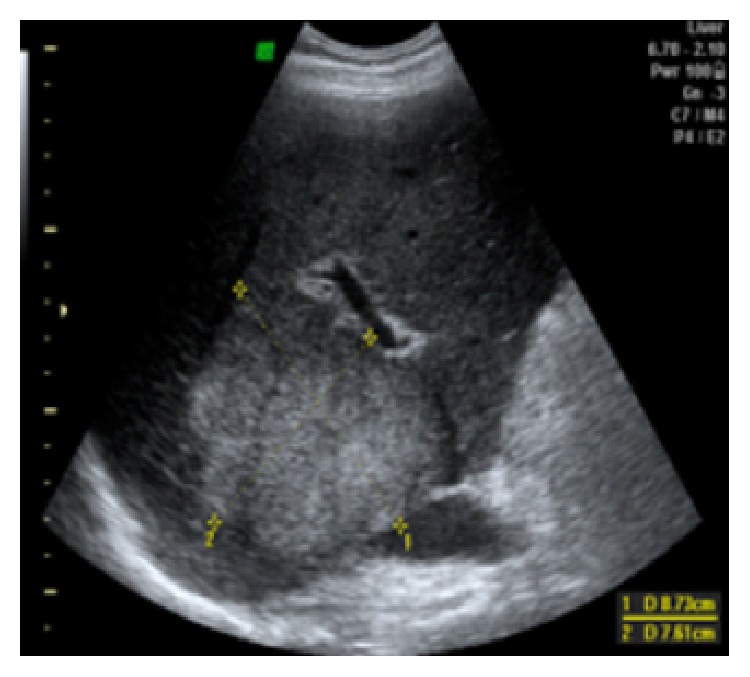
US of the liver after electric shock. US image of the liver shows the presence of hyperechogenic, homogenous, nonlinearly shaped focal lesion, situated in segments VIII and V, sized 7 cm × 8 cm.

**Figure 2:**
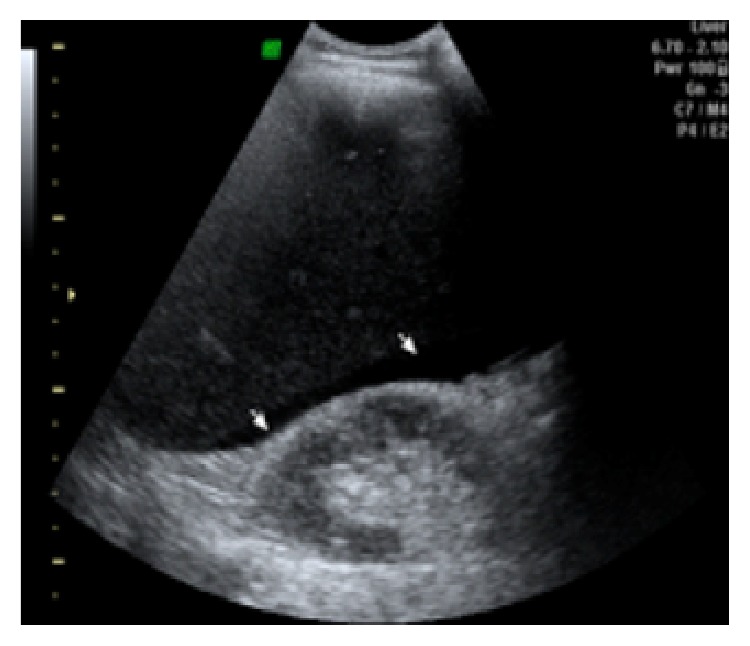
US image shows small collection of fluid in hepatorenal recess.

**Figure 3:**
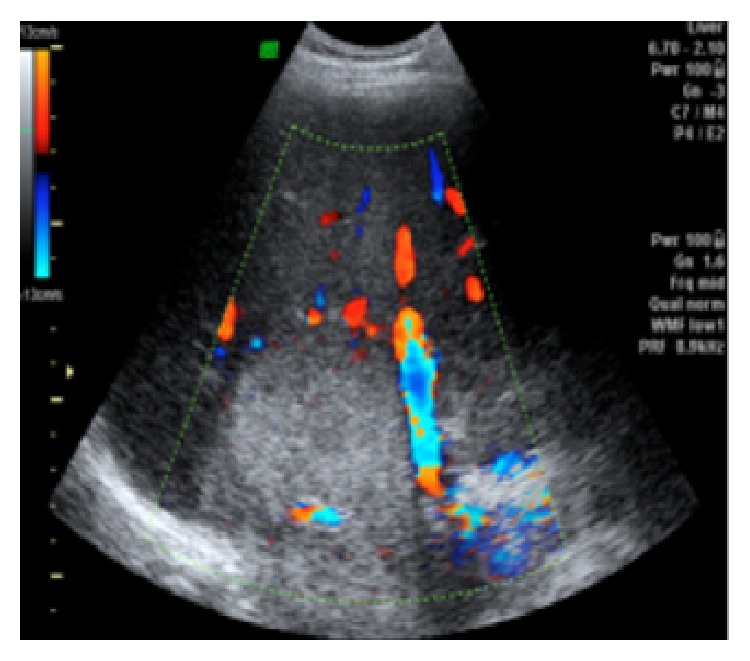
Color Doppler shows no signs of vascularization centrally, with only slight signs of vascularization around periphery.

**Figure 4:**
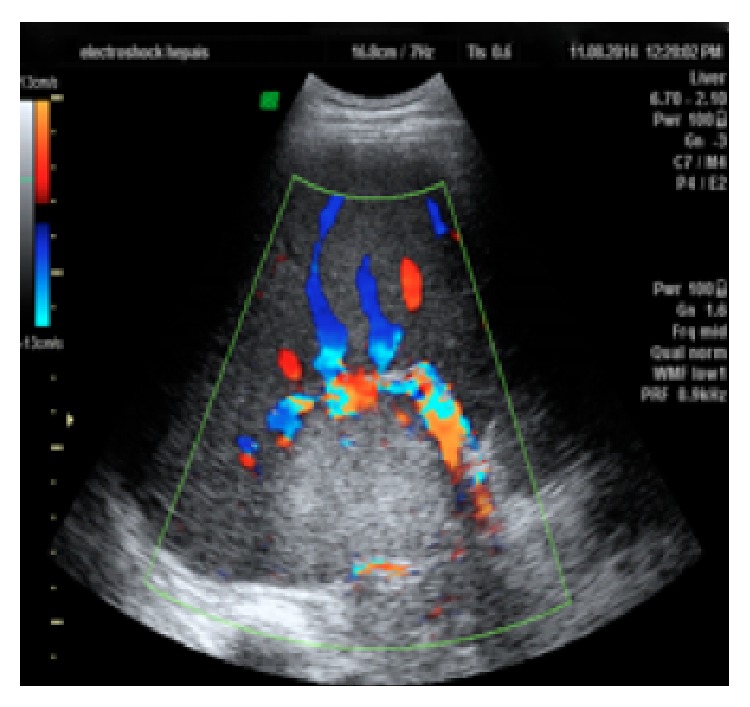
Color Doppler shows no signs of vascularization centrally, with only slight signs of vascularization around periphery.

**Figure 5:**
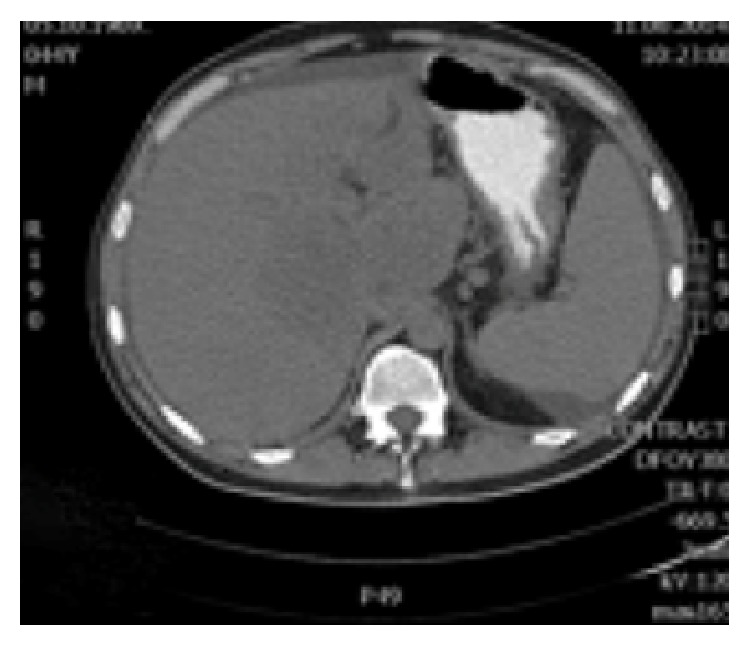
On noncontrast CT phase lesion was poorly visualized, shaped as a flower with a lower density than liver parenchyma.

**Figure 6:**
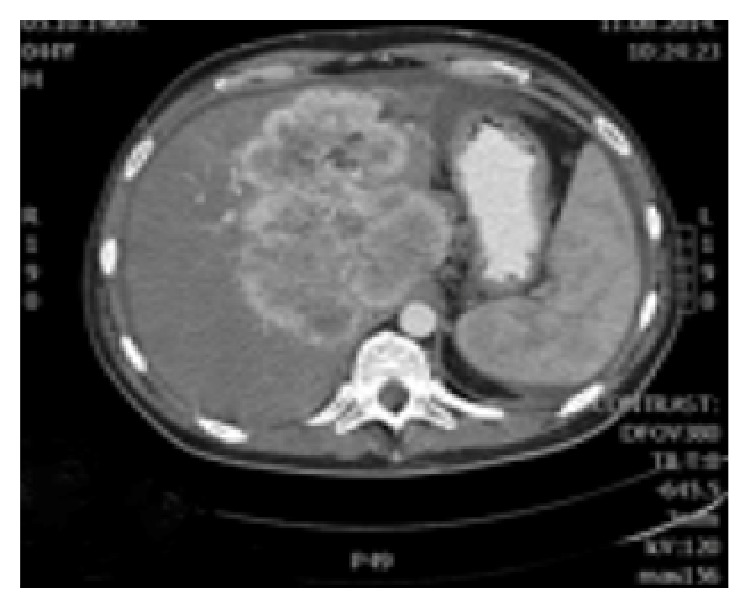
In arterial phase liver lesion was better visualized. Central parts of the lesion after the contrast has been administered remained hypodense, while margins of the lesion were intensively hyperdense.

**Figure 7:**
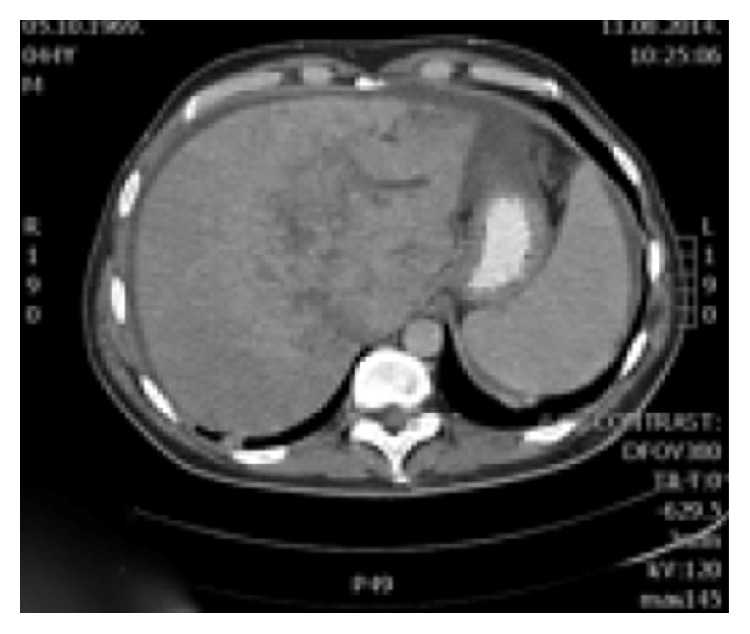
In portal venous phase, peripheral part of the liver lesion became washed out, except in the central hypodense parts.

A 39-year-old male with skin burns on his right arm and right hemiabdomen has been referred from the emergency room. Injuries were caused by contact with high voltage (220 kV) electrical current via fishing rod he held in his right hand. Lab tests upon arrival showed high values of serum bilirubin and low values of iron, while ECG and other lab results had normal values. Initial liver ultrasound, done one hour after the electric shock, was normal (GE, USA). However, due to the unstable condition and severe burns on the patient's right hemiabdomen, the first ultrasound was limited in time, quality, and subjectivity of the radiologist. This would explain why there was no substantial tissue destruction visualized with the first ultrasound, as expected in the liver injury caused by Joule heating. A day after, a follow-up abdominal ultrasound showed an extensive liver damage, which presented on the ultrasound as a hyperechogenic, homogenous, nonlinearly shaped focal lesion, situated in segments VIII and V, sized 7 cm × 8 cm (Figure 1). In hepatorenal recess a small collection of fluid was observed (Figure 2). Doppler imaging in the described area did not show signs of vascularization centrally, with only slight signs of vascularization around periphery (Figures 3 and 4). US was followed with CT of the abdomen, done on multilayered Siemens apparatus (Erlangen, Germany) in three phases: noncontrast phase, arterial phase, and portal venous phase. On noncontrast CT phase lesion was poorly visualized, shaped as a flower with a lower density than liver parenchyma (11–50 HJ) with larger dimensions, 11 cm × 14 cm (Figure 5). In arterial phase liver lesion was better visualized. Central parts of the lesion after the contrast has been administered remained hypodense (23 HJ), while margins of the lesion were intensively hyperdense, up to 116 HJ (Figure 6). In portal venous phase, peripheral part of the liver lesion became washed out, except in the central hypodense parts. This liver lesion was marked as focal coagulant necrosis (Figure 7).

## 3. Discussion

Electric shock can occur upon a contact of human body with high voltage direct or alternate current, causing traumatic blunt injury.

Because of the muscle tetany, a respiratory failure due to respiratory muscle paralysis can happen, as well as ventricular fibrillation which can cause a heart attack. Pathogenesis and pathophysiology of electrical injuries are more complex than previously thought [6]. Pathophysiology of electrical injury of internal organs is still unclear, probably due to the large number of variables that cannot be measured when the high voltage electricity passes through the tissue. It seems that the injury is of thermogenic nature and most of histological studies show coagulation necrosis [7]. The increase in temperature causes irreversible denaturation of macromolecules [8]. Electroporation can cause cell necrosis in the absence of heat. It is believed that due to electric discharges in tissue the effect of electroporation occurs as well as changes in configuration of proteins that threaten the integrity and walls of the cell [9–11].

A contact with alternate current is three times more dangerous than a contact with direct current of the same voltage. Muscle tetany that results can cause respiratory arrest because of the paralysis of respiratory muscles or ventricular fibrillation. Electricity applied in domestic households can cause heart arrest. First artificial electricity related injuries were reported 300 years ago. The first recorded accidental electrocution occurred in 1879 when a stage carpenter in Lyon, France, touched a 250-volt AC generator. Since then, worldwide, many electrocutions have been reported, but there are a limited number of articles that discuss US and CT diagnostic imaging of damaged liver caused by electrocution.

Pathophysiology of electrical injury of visceral organs still has not been understood, probably because of number of variables that cannot be measured when high voltage electricity goes through tissue. It seems that the injury is of thermal nature and most histologic studies reveal electrothermal coagulation necrosis. It is believed that due to electric discharges in tissue the effect of electroporation occurs as well as changes in configuration of proteins that threaten the integrity and walls of the cell. The nature and severity of electrical burns are directly proportional to the power, resistance, and duration of the current passing through the body. Human tissue as a specific material has a trait of electrical resistance: it resists the flow of electricity. The greater the resistance of the tissue, the greater the possibility of transformation of electric energy into thermal energy. The amount of resistance depends on the specifics of a particular tissue, depending on the moisture content, temperature, and other physical properties.

Lee and Kolodney have studied the thermal response of the human upper extremity to large electric currents using an axisymmetric unidimensional model containing bone, skeletal muscle, fat, and skin in coaxial cylindrical geometry and have found that when the tissues are electrically in parallel, skeletal muscle sustained the largest temperature rise and then heated adjacent tissues. Thus, when bone is not in series with other tissues, Joule heating of bone is unlikely to be responsible for thermal damage to adjacent tissue. In addition, the effect of tissue perfusion on the thermal response was found to be essential for rapid cooling of the centrally located tissues [12].

Primary resistance of the body to the flow of electricity is skin. It is not possible to predict the amount of underlying tissue damage based on the amount of cutaneous involvement. Remaining visceral organs, including the liver, provide resistance indirectly. When it comes to liver, blunt traumatic injury occurs from a focal hepatic necrosis coagulation and is linked to development of coagulopathy of coagulation factors V and X.

Victims of electric shock are generally not able to provide adequate information on the occurrence of injuries. The most of them are in a state of shock and hypoxia in severe cases or state of unconsciousness and confusion in milder injuries.

Visible injuries are at the point of entry and exit from the electricity, and the severity of injuries of other organs is generally disproportionate to the surface of the body burned [13, 14]. Visceral lesions are rare but potentially serious and require to be properly managed by a multidisciplinary team [15].

Diagnostic imaging is indicated for patients after the electric shock with suspected damage to the viscera. Depending on the clinical picture additional tests and examinations are done. To prevent kidney and heart failure, electrocardiographic, hemodynamic, and intra-abdominal pressure monitoring is essential, as is maintaining water balance and proper management of rhabdomyolysis.

Thorough physical examination combined with diagnostic imaging and laboratory tests allow an early diagnosis of serious injury and early intervention, which reduces morbidity and mortality.

## 4. Conclusion

In addition to the visible thermal skin injuries, electric shock can make thermal and coagulation based injuries to liver and other organs due to resistance to the flow of electric current. Besides physical exam and laboratory tests, US and CT are essential in early diagnosis of electrical injuries, allowing an early intervention, which results in reduction in mortality and morbidity.
